# Scans per day as predictors of optimal glycemic control in people with type 1 diabetes mellitus using flash glucose monitoring: what number of scans per day should raise a red flag?

**DOI:** 10.1007/s00592-023-02204-x

**Published:** 2023-11-06

**Authors:** Fernando Sebastian-Valles, Julia Martínez-Alfonso, Jose Alfonso Arranz Martin, Jessica Jiménez-Díaz, Iñigo Hernando Alday, Victor Navas-Moreno, Teresa Armenta Joya, Maria del Mar Fandiño García, Gisela Liz Román Gómez, Luis Eduardo Lander Lobariñas, Purificación Martinez de Icaya, Miguel Antonio Sampedro-Nuñez, Vicente Martínez-Vizacaíno, Mónica Marazuela

**Affiliations:** 1 Department of Endocrinology and Nutrition, Hospital Universitario de La PrincesaInstituto de Investigación Sanitaria de La Princesa, Universidad Autónoma de Madrid, 28006 Madrid, Spain; 2 Department of Family and Community Medicine, Hospital La Princesa/Centro de Salud Daroca, 28006 Madrid, Spain; 3 Department of Endocrinology and Nutrition, Hospital Universitario Severo Ochoa, 28194 Leganés, Madrid, Spain; 4 Department of Endocrinology and Nutrition, Hospital Universitario Basurto, 48013 Bilbao, Spain; 5 Health and Social Care Research Center, Universidad de Castilla-La Mancha, 16071 Cuenca, Spain; 6 Facultad de Ciencias de la Salud, Universidad Autónoma de Chile, Talca, Chile

**Keywords:** Type 1 diabetes, Flash glucose monitoring, Continuous glucose monitoring systems, Optimal control, Real world data

## Abstract

**Aims:**

This study aimed to determine the minimum frequency of flash glucose monitoring (FGM) scans necessary for optimal glycemic control in patients with type 1 diabetes (T1D).

**Methods:**

Data were collected from 692 patients (47.5% female, with a median age of 47.4 years) who used FGM systems daily and recorded their clinical variables and device data.

**Results:**

Logistic regression models showed that performing more than 12 scans per day was associated with improved T1D control (OR = 4.22, *p* < 0.001) and a reduction in HbA1c (7.6 vs 7.0%, 60–53 mmol/mol *p* < 0.001). However, those performing less than 6 scans showed no improvement in HbA1c (7.9 vs 7.8%, 63–61 mmol/mol *p* = 0.514). Thirteen daily scans were determined as the optimal cutoff point for predicting optimal glycemic control using a maximally selected rank algorithm. Significant reductions were observed in mean glucose (< 0.001), coefficient of variation (< 0.001), HbA1c (< 0.001), and an increase in TIR (< 0.001) in patients who performed more than 12 daily scans.

**Conclusions:**

The results suggest that a higher frequency of daily scans by T1D patients using FGM systems leads to improved chronic glycemic control. The minimum recommended frequency for optimal control is 13 scans per day, and more than 6 daily scans are needed to improve HbA1c.

**Supplementary Information:**

The online version contains supplementary material available at 10.1007/s00592-023-02204-x.

## Introduction

Type 1 diabetes mellitus (T1D) requires accurate and frequent glucose measurements for proper metabolic control and prevention of complications [1]. Nowadays, the development and advances in continuous glucose monitoring (CGM) systems have been a substantial step forward in the monitoring and treatment of diabetes mellitus (DM) due to the simplicity of self-testing and the quality of the information, which has eventually led to improved disease management [2–4]. In this sense, the widespread access of patients with T1D to CGM devices can provide new insights into the determinants of disease control, given the large number of patients using these systems, particularly flash glucose monitoring (FGM) systems, and the enormous amount of data they provide.

The assessment of chronic control using glycated hemoglobin (HbA1c) is considered a less accurate measure than continuous interstitial glucose monitoring, either real-time (rt-CGM) or flash (FGM), to determine the optimal approach for patient management [5–7]. In addition, HbA1c does not allow an appropriate assessment of glycemic variability, time spent in hypoglycemia or within the glucose range, which are becoming increasingly important in clinical practice [8, 9]**.**

Although capillary blood glucose self-testing provides very useful information, one of its main limitations [10] is its highly variable adherence [11, 12]. Regarding CGM, one of the issues that remain to be elucidated is the impact of daily scanning frequency. The International Consensus on Time in Range indicates that at least 70% of sensor usage time is necessary for reproducible data and clinical benefit from the use of this monitoring [13], although some studies have recently suggested that more time is probably needed to obtain the best performance from these sensors [14, 15]. Nonetheless, the importance of the frequency of scanning has been evaluated in children [16, 17] but remains to be assessed in adults.

Thus, this study aimed to assess the influence of daily FGM scanning on the chronic control of T1D as measured by both HbA1c and glycemic parameters provided by FGM systems and to estimate a cutoff point of number of daily scans for clinical benefits. Moreover, we aimed to validate the performance of this cutoff point by testing whether patients who are above this cutoff point have clinically significant improvements in glycemic control parameters.

## Materials and methods

This is a retrospective follow-up study including 1135 patients who were regular users of the FreeStyle Libre® (Abbott) FGM system between July and August 2022 at two hospitals in Madrid, Spain. Patients diagnosed with T1D, cystic fibrosis-related diabetes (CFRD), and pancreatic diabetes were included. Patients with a diagnosis of type 2 diabetes (T2D) or MODY, those with a usage time of less than 70%, and those who did not have a download of sensor data in the 30 days before data collection were excluded. The final sample consisted of 692 patients. FGM data of these patients were retrieved from days 14 and 90 of follow-up.

### Procedures

Prior to starting to use the FreeStyle monitor, all patients received a training session on the use of the monitor according to international recommendations [13]. The system consists of a glucose oxidase–based electrochemical sensor, which is placed subcutaneously and replaced every 14 days, along with a receiver to which interstitial glucose measurements are sent wirelessly and stored in the cloud using the Libreview platform. All patients were provided with written instructions on how to use the data provided by FGM to make real-time adjustments of insulin doses and on the use of Libreview cloud to retrospectively review the glucose data to adjust future insulin doses. All patients were instructed to modify their insulin doses and treatment of hypoglycemia based on their glucose trend.

### Data collection

Data including sociodemographic and clinical details, as well as laboratory tests and pharmacologic medication for T1D, were obtained from electronic health records. Glucometric information cloud downloads from the Libreview platform using the FreeStyle 2 device (FreeStyle Libre 2®, Abbott) were retrieved at 14 and 90 days. We collected the following variables: time in range (TIR), time below and above range (glycemia < 70 mg/dL or > 180 mg/dL, respectively), number of daily readings, sensor usage, hypoglycemia events, coefficient of variation (CV) and standard deviation (SD). In addition, sociodemographic and clinical data were collected, including sex, age, duration of diabetes mellitus, type of diabetes, body mass index (BMI), smoking, continuous subcutaneous insulin infusion (CSII) carrier, baseline HbA1c (immediately before the sensor was placed), last available HbA1c, FGM usage time, time of disease evolution, age at disease onset, insulin dose, and retinopathy. Glycated hemoglobin was routinely determined using liquid chromatography (ADAMS A1c HA8180 V ARKRAY®).

All patients included in the study were informed of its objectives and accepted the use of their medical history data for research purposes. The Research Ethics Committee of Hospital de La Princesa, Madrid approved this study (Study number: 2022-4997-17/22).

### Statistical analysis

The statistical analysis was performed using R version 4.0.3 [18] and STATA 17.0 statistical software. After checking the plausibility of outliers, the fitting to a normal distribution was examined using both statistical (Kolmogorov‒Smirnov test) and graphical (normal probability plot) procedures. Those variables with extreme values for which their authenticity was questionable were winsorized using the 99th and 1st percentiles of the distribution. Continuous variables are presented as the mean and standard deviation (SD), and categorical variables are presented as numbers and percentages of the samples. We calculated a dichotomous optimal glycemic control variable as time in range > 70% and time below range (< 70 mg/dL) < 4%, as recommended [13].

Bivariate differences were tested using Student’s *t* test and the Mann‒Whitney *U* test, depending on their adjustment to a normal distribution. Logistic regression models were estimated using HbA1c < 7% (53 mmol/mol) and optimal glycemic control as dependent variables, glycemic control variables (time in range, time spent in hypo- and hyperglycemia, hypoglycemia events), number of daily readings and sensor usage as independent variables, and sociodemographic and clinical variables as covariates. The MaxStat package of R was selected using maximally selected rank algorithm to identified the optimal cutoff point for the number of scans/days to achieve optimal glycemic control [19]. To validate the use of this cutoff point, we examined its influence on the chronic control of patients with T1D by testing mean differences in glycemic control parameters between those above and below this cutoff point. Moreover, analysis of variance was used to test the effect of daily scans frequency categories (3–6, 7–9, 10–12 and > 12) on pre/post HbA1c changes.

## Results

### Characteristics of the patients

Table 1 describes the characteristics of the study sample. After removing the 443 individuals who did not meet the inclusion criteria, data from 692 patients (47.5% females) aged between 18 and 89 years (mean 47.4, SD 15.5) were analyzed. Type 1 diabetes was the most frequent diagnosis (94.1% of the users), followed by pancreatic diabetes (4.43%) and cystic fibrosis-related diabetes (1.48%). The mean age at disease onset was 25.7 (SD 16.6) years, and the mean time of disease duration was 21.7 (SD 13.6) years. The mean duration of FGM FreeStyle Libre® usage (years) was 1.8 (SD 1.1). Of the study sample, 94.7% of patients were users of multiple doses of insulin in a bolus-basal strategy, and 5.4% were users of open loop CSII. HbA1c prior to FGM placement was 7.7 (± 1.29) % (61 ± 14.4 mmol/mol).

**Table 1:** Baseline characteristics of the study sample

Variable	Obs *n* = *692*
Age	47.4 (± 15.5)
Sex, women (%)	329 (47.5)
Type 1 diabetes	653 (94.1)
Age debut (years)	25.7 (± 16.6)
Multiple daily injections (%)	655 (94.7)
Insulin pump (CSII) (%)	37 (5.4)
BMI (Kg/m^2^)	25.5 (± 4.24)
Smokers (%)	129 (18.6)
Duration of diabetes (years)	21.7 (± 13.6)
User time FGM (years)	1.8 ± (1.1)
Mean pre-FGM HbA1c (%, mmol/mol)	7.7 ± 1.29 (61 ± 14)
HbA1c (%,mmol/mol)	7.3 ± 1.09 (56 ± 12)
Optimal glycemic control (%)	175 (25.3)
Insulin (UDS)	42.5(± 18.94)

Data are mean (± SD) or *n* (%). Optimal glycemic control is operationally defined as spending more than 70% of the time within the range of 70–180 mg/dL and less than 4% of the time below 70 mg/dL, utilizing data obtained from a 14-day sensor download

*FGM* flash glucose monitoring

### Glycemic control

**Fig. 1:**
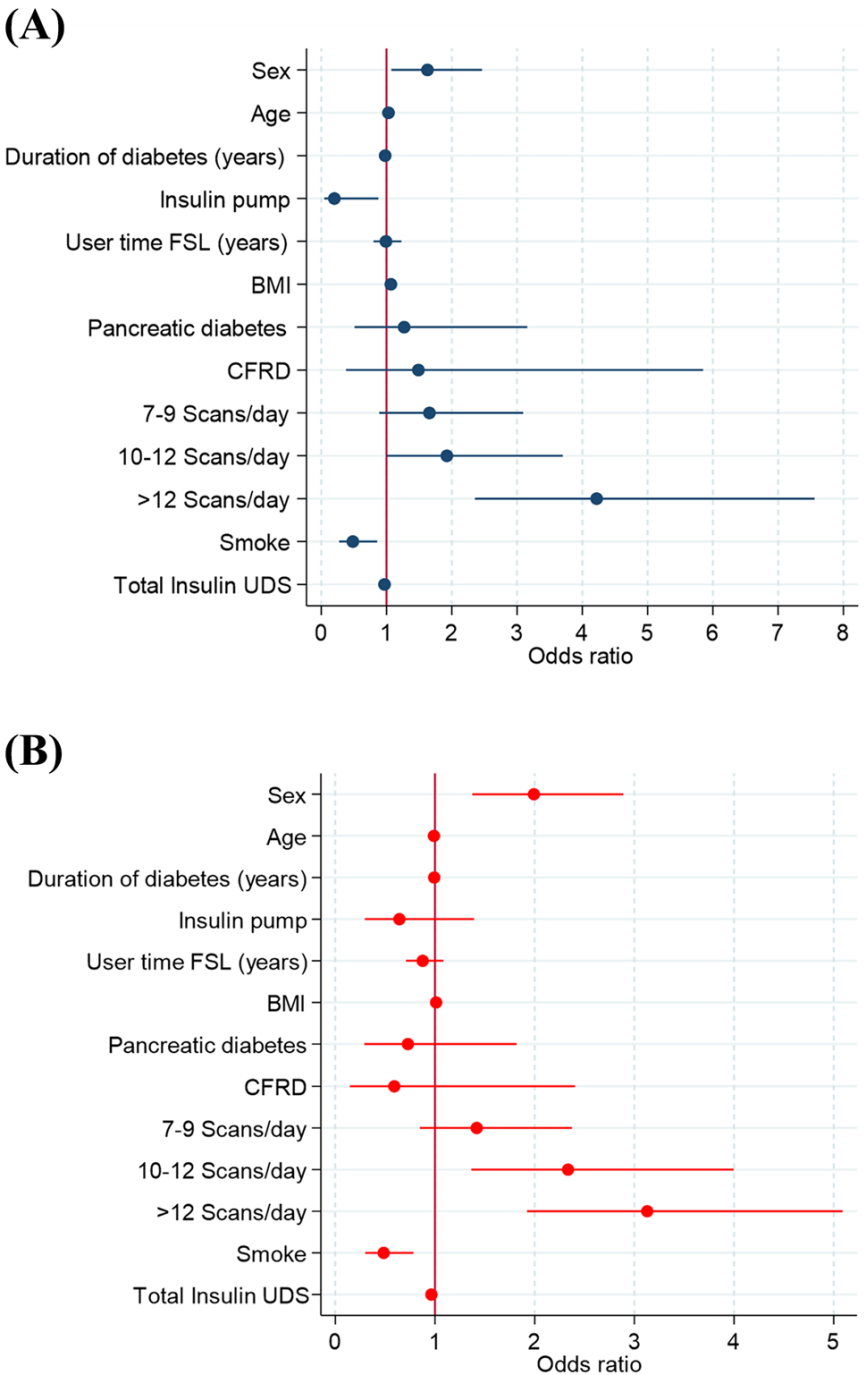
Predictors of optimal glycemic control (**A**) and HbA1c < 7% (**B**) BMI: body mass index CFRD: cystic fibrosis-related diabetes DM3c: diabetes mellitus secondary to chronic pancreatitis and pancreatic cancer. More than 12 scans per day was the strongest predictor of optimal control in flash glucose parameters OR = 4.22 (*p* < 0.001) and HbA1c OR = 3.13 (*p* < 0.001)

Number of scans (> 12) OR = 4.22 (*p* < 0.001), smoking OR = 0.48 (*p* = 0.013), male sex OR = 1.63 (*p* = 0.022), age OR = 1.03 (*p* < 0.001), time of disease progression OR = 0.98 (*p* = 0.009), BMI OR = 1.07 (*p* = 0.03), use of open loop CSII OR = 0.20 (*p* = 0.033) and total daily insulin dose (TDD) OR = 0.97 (*p* < 0.001) were significant predictors of optimal glycemic control in a logistic regression model using the FGM data. Time as device user and type of DM were not independent predictors of good control (Fig. 1A).

When HbA1c < 7% (53 mmol/mol) was used as the glycemic control variable, > 12 daily readings were also the strongest independent predictor of glycemic control, OR = 3.13 (*p* < 0.001). Both male sex (OR = 1.99, *p* < 0.001) and smoking (OR = 0.49, *p* = 0.003) were also predictors of glycemic control estimated with HbA1c (Fig. 1B).

In the analysis of the 90-day FGM data, a scanning frequency equal to or greater than 12 readings per day remained a strong predictor of optimal glycemic control (OR = 3.1, *p* < 0.001). Age (OR = 1.03, *p* < 0.001), BMI (OR = 1.09, *p* = 0.03), duration of diabetes (OR = 0.97, *p* < 0.001) and TDD (OR = 0.98, *p* = 0.006). However, sex and smoking were not statistically significant independent factors (*p* = 0.061 and *p* = 0.306, respectively). (Figure S1) (Supplementary Data S1).

### Cutoff point

The assessment of the cutoff point of a minimal number of readings for optimal glycemic control in T1D is depicted in Figure S2 (Supplementary Data S2). After obtaining a cutoff point of 13 readings, differences in glycemic parameters were tested between those who read sensor data more than 12 times and those who did not. Table 2 shows a significant reduction in mean blood glucose 167.5 to 147.5 mg/dL (< 0.001), coefficient of variation 36.8 to 32.8% (< 0.001), and HbA1c 7.5 to 7.0% (58.5–53 mmol/mol)(< 0.001) and a significant increase in the TIR 59.0 to 71.5% (< 0.001) in patients with more than 12 daily readings. No statistically significant differences were observed in time below range (< 70 mg/dL) (TBR) (*p* = 0.134).

**Table 2:** Glycemic parameters in scan frequency groups according to the 13 scans/day cutoffpoint

Variable	< = 12 scans/day	> 12 scans/day	Effect size (CI 95%)	*p* value
*n*	486	206		
Time in Range (%)	59.0 (± 18.0)	71.5 (± 14.9)	− 0.73 (− 0.90, − 0.56)	< 0.001
HbA1c (%, mmol/mol)	7.5 ± 1.1 (58 ± 12)	7.0 ± 1.0(53 ± 11)	0.46 (0.29, 0.62	< 0.001
Coefficient of variation (%)	36.8	32.8	0.59 (0.42, 0.76)	< 0.001
Time below range (< 70 mg/dL) (%)	4.4	3.8	0.12 (-0.04, 0.28)	0.134
Mean Glucose, mg/dL (SD)	167.5 (± 38.3)	147.5 (± 25.3)	0.57 (0.41, 0.74)	< 0.001
Time above range (> 180 mg/dL) (%)	36.6	24.6	0.65 (0.48, 0.82)	< 0.001
Optimal glycemic control	79 (17.8)	97 (39.0)	− 0.41 (− 0.53, − 0.28)	< 0.001

Comparison of glycemic parameters and HbA1c between the low-frequency group and high-frequency group. Data are percentages except for sample size of group (*n*) and for mean glucose. Glycemic parameters are presented in mg/dL. Optimal glycemic control is defined as time in range > 70% and time below range < 70 mg/dL) < 4%. The group with more frequent scanning had better time above range, mean glucose, a coefficient of variation range than the group with less scanning

### Impact of daily scans on HbA1c improvement

**Fig. 2:**
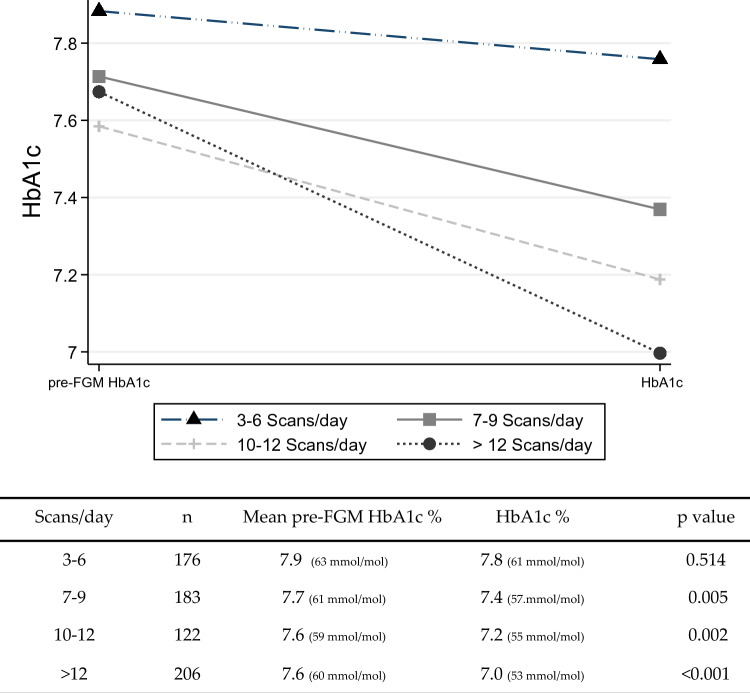
Influence of the number of sensor readings on HbA1c change after FGM placement. FGM: flash glucose monitoring. Patients who performed more than 12 daily scans per day showed an improvement of 0.65% (*p* < 0.001) in HbA1c. The groups of 6–9 and 9–12 daily scans also improved HbA1c by 0.36% (*p* = 0.005) and 0.39% (*p* = 0.002), respectively. However, no statistically significant improvement was observed in the group with fewer than six daily readings

When studying the effect of daily readings on changes in HbA1c values by comparing pre/post sensor use HbA1c values (Fig. 2), an improvement of 0.6% (7 mmol/mol) (*p* < 0.001) in HbA1c was observed in the group with more than 12 daily scans. The groups of 7–9 and 10–12 daily scans also improved HbA1c by 0.4% (4 mmol/mol) (*p* = 0.005) and 0.3% (3 mmol/mol) (*p* = 0.002), respectively. However, no statistically significant improvement was observed in the group with fewer than six daily readings.

## Discussion

Our study aimed to examine the influence of FGM scanning frequency on chronic control of T1D and to estimate and validate a cutoff point for the minimum number of scans per day for optimal glycemic control. Our data support that thirteen is the minimum number of scans per day that provides better glycemic parameters and optimized glycemic control for these patients.

The usefulness of rt-CGM and FGM systems in the management of DM is currently unquestionable [20–23]. A reduction in up to 0.6% in HbA1c in patients with MDI (multiple insulin doses) using CGM compared to those using capillary blood glucose has been reported in randomized clinical trials [24, 25]. Moreover, improvements in other glycemic control parameters such as time in range or glycemic variability [15, 26], in both T1D [27] and T2D patients have been observed [28]. However, although most literature supports a beneficial effect on DM chronic control as assessed by HbA1c, issues such as the appropriate use of CGM have been rarely studied.

A potential association between the number of daily FGM scans and improvement of glycemic parameters was assessed by logistic regression. Controlling for other potential confounders, such as age, biological sex, BMI, smoking, age at onset and time from the diagnosis of T1D, our logistic regression model supports, in accordance with previous evidence [14, 29, 30], that the number of daily scans is directly associated with optimal glycemic control as assessed by both the sensor’s glycemic indicators and HbA1c value.

Moreover, data from these models, as well as ROC curves for determining the cutoff point for optimal glycemic control, estimated that more than twelve is the appropriate number of daily scans needed for better control of diabetes. This scanning frequency is greater than that recommended by the American Diabetes Association (up to one scan every 8 h) for the appropriate use of these devices. In addition, in our analyses, when comparing HbA1c data prior to device placement with the last HbA1c value available, patients who performed less than 6 daily scans did not improve their HbA1c control despite using the sensor within the international consensus recommendation of time in range (use > 70% with updated data) [13].

This cutoff point was further confirmed by two analysis strategies: the mean glycemic parameters were compared between those who met this mean number of scans/day and those who did not, and the change in HbA1c value at sensor placement was compared to the most recent one (median 1.8 years follow-up). Both the main glycemic parameter levels and HbA1c values were substantially better among those who performed more than 12 scans/day than among those who did not (Table 2). These results, together with cutoff curve analyses, support that optimal FGM use to improve glycemic control requires patients to scan FGM devices more than 12 times a day. However, it is difficult to assess the consistency of our estimates of the number of daily scans for optimal use of FGM devices because no similar study has been published thus far, with the exception of a study in children reporting an association between the number of scans per day and glycemic control [16, 17].

The observed beneficial effect of glucose readings on glycemic control may be attributed to an increased number of scans, indicating heightened awareness of blood glucose levels and enhanced quality and quantity of glycemic information. Nevertheless, the most crucial aspect of CGM lies in monitoring glucose trends and adjusting insulin dosages based on CGM data, facilitating better therapeutic decision-making and preventing hypoglycemia, a central objective in diabetes management [31].

In this context, our study did not observe any differences in TBR between the group with higher readings and the group with lower readings. One plausible explanation could be that the FreeStyle 2 system provides alarms to alert patients when they reach the TBR threshold, which might account for the low TBR rates in both groups (TBR 3.8 vs 4.4%). Consequently, this makes it challenging to identify statistically significant differences between the two groups.

Although in our study, the daily number of sensor scans was the variable most strongly associated with good glycemic control, associations have also been observed in non-smokers and males. The hyperglycemic effect of tobacco has been demonstrated in both T1D [32] and T2D [33] and appears to be mediated by the mTOR pathway [34]. On the other hand, in our study, female sex was associated with not achieving glycemic targets, which has been previously reported in studies with large patient samples [35, 36] However, none of these associations were observed in the sensor data analysis over 90 days.

Lastly, younger age, longer diabetes duration, and higher daily insulin requirements were consistently associated with poorer chronic control, as indicated by both glycated hemoglobin and sensor data at 14 and 90 days. These findings are consistent with results from a large cohort study conducted in 75 centers in our country [37], where similar outcomes were observed.

This study has certain limitations that should be acknowledged. First, it is an observational follow-up study that does not allow to compare the efficacy in improving glycemic control between patients with and without FGM devices. Moreover, although we controlled for numerous covariates in the analyses, some potential confounders that we were unable to control, such as dietary habits, level of diabetes education or socioeconomic status, could influence the validity of our results. Secondly, the inclusion criteria, where patients with a high usage time of FGM (> 70%) and regular download of sensor data were included, could potentially introduce bias in the scan frequency. Third, the two centers from which the patients were enrolled are located in different cities, within the same region, so our conclusions may not be completely extended to overall patients with T1D. Forth, the effect on long-term glycemic control was not evaluated; thus, prospective studies will be necessary to evaluate the long-term effect of daily scanning frequency on glycemic control parameters.

Another limitation of our study is not having analyzed the time in tight range, (the time when glucose readings are within 70–140 mg/dL or 3.9–7.8 mmol/L), which is an emerging variable as an informed measure of time in range, particularly for individuals with type 1 diabetes using automated insulin delivery systems or individuals with type 2 diabetes using glucose-lowering agents [13].

Last, glycemic parameters are commonly used because there is consistent evidence supporting that optimum control of these indicators delays the onset of diabetic complications. However, only long-term follow-up studies comparing the incidence of these complications in those who adequately use FGM devices and those who do not would be able to provide solid evidence as to whether the use of these devices could delay the onset of diabetic complications.

In conclusion, our study supports the notion that increased daily scanning frequency using FGM systems leads to improved glycemic control in individuals with T1D. Our findings suggest that a minimum of 13 scans per day could be necessary to achieve optimal control, while a frequency greater than 6 scans per day can result in improved HbA1c levels.

### Supplementary Information

Below is the link to the electronic supplementary material.

Supplementary file1 (DOCX 140 kb)

## Data Availability

F.S.-V and M.M. are the guarantor of this work and, as such, had full access to all the data in the study and takes responsibility for the integrity of the data and the accuracy of the data analysis.
