# Supplementary material for: Scans per day as predictors of optimal glycemic control in people with type 1 diabetes mellitus using flash glucose monitoring: what number of scans per day should raise a red flag?
Source: Acta Diabetol. 2023 Nov 6;61(3):343–50. doi: 10.1007/s00592-023-02204-x (PMC10948530; doi:10.1007/s00592-023-02204-x)

SUPLEMENTARY DATA S1

Figure S1

Predictors of flash glucose monitoring optimal control (time in range >70% and time below range) <4% in 90 days data.


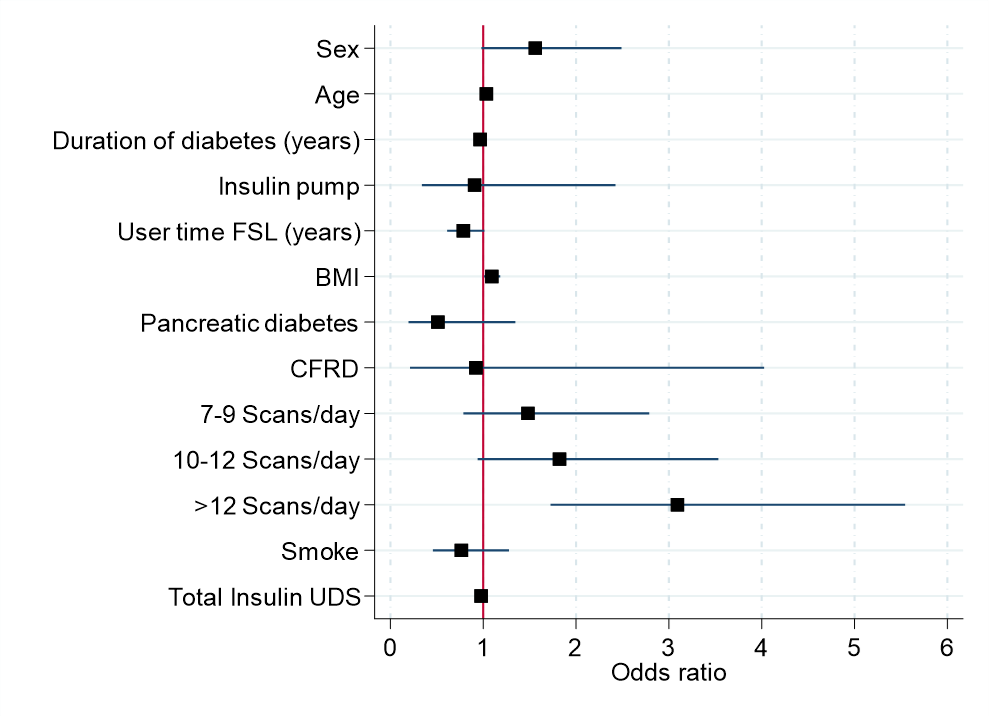


BMI: Body Mass Index CFRD: Cystic Fibrosis Related Diabetes DM3c: diabetes mellitus secondary to chronic pancreatitis and pancreatic cancer. More than 12 readings per day remained a strong predictor of optimal glycemic control (OR= 3.1, p<0.001).

SUPLEMENTARY DATA S2

**Figure S1**

MaxStat optimal cutoff point for the number of scans/days to achieve optimal glycemic control.

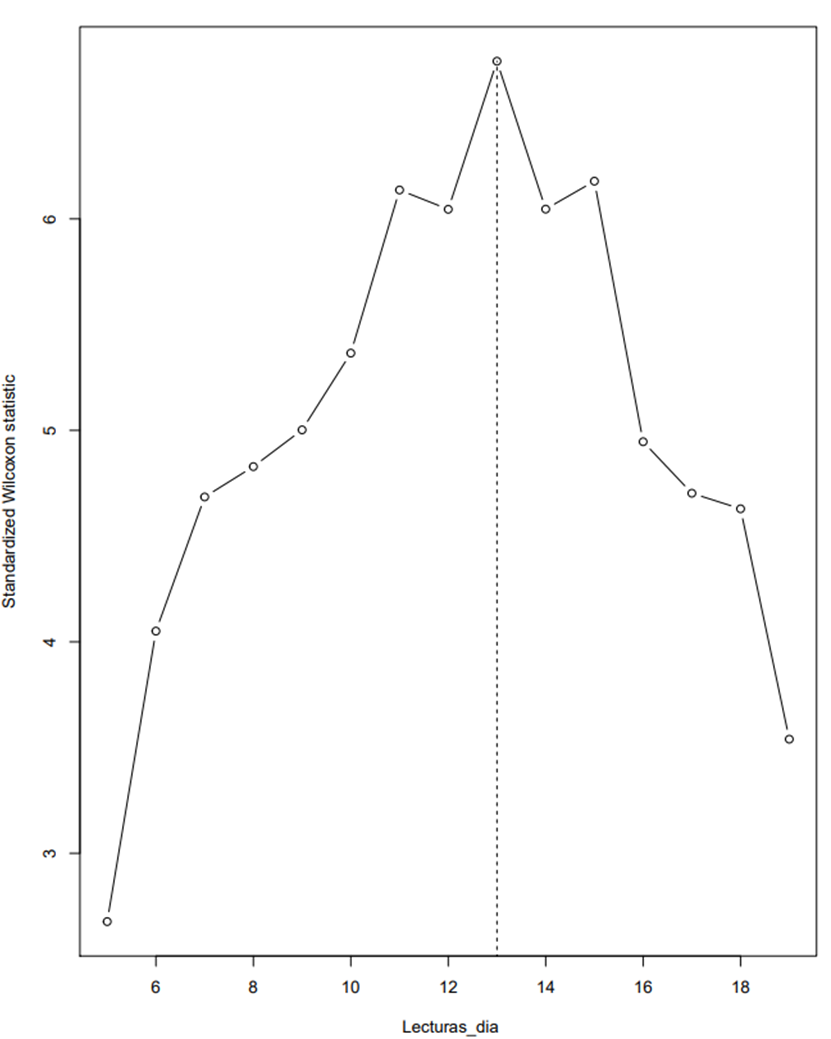

Supplement: Supplementary file 1 — Supplementary file1 (DOCX 140 kb) [file 592_2023_2204_MOESM1_ESM.docx]
